# ICTV Virus Taxonomy Profile: Phasmaviridae 2024

**DOI:** 10.1099/jgv.0.002002

**Published:** 2024-07-03

**Authors:** Jens H. Kuhn, Holly R. Hughes

**Affiliations:** 1 Integrated Research Facility at Fort Detrick, Division of Clinical Research, National Institute of Allergy and Infectious Diseases, National Institutes of Health, Fort Detrick, Frederick, MD 21702, USA; 2 Centers for Disease Control and Prevention, Division of Vector-Borne Diseases, Fort Collins, CO 80521, USA

**Keywords:** ICTV Report, orthophasmavirus, *Phasmaviridae*, phasmavirus, taxonomy

## Abstract

*Phasmaviridae* is a family for negative-sense RNA viruses with genomes of about 9.7–15.8 kb. These viruses are maintained in and/or transmitted by insects. Phasmavirids produce enveloped virions containing three single-stranded RNA segments that encode a nucleoprotein (N), a glycoprotein precursor (GPC), and a large (L) protein containing an RNA-directed RNA polymerase (RdRP) domain. This is a summary of the International Committee on Taxonomy of Viruses (ICTV) Report on the family *Phasmaviridae,* which is available at ictv.global/report/phasmaviridae.

## Virion

**Fig. 1.**
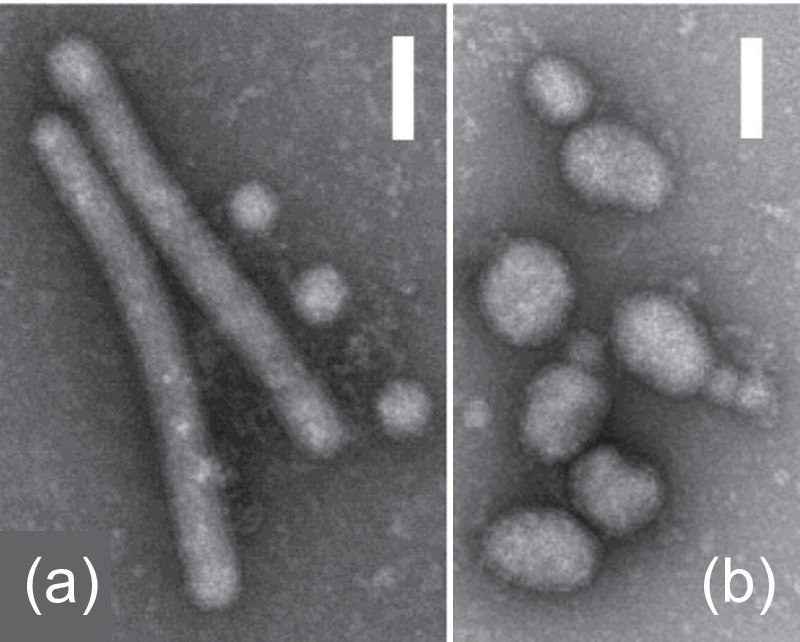
Electron micrograph of negative-stained virions of jonchet virus (**a**) and Ferak virus (**b**) sedimented by ultracentrifugation. Scale bars: 100 nm (images reproduced from [1]).

Phasmavirids produce virions that are spherical or pleomorphic (60–120 nm in diameter) or tubular (60×600 nm), with lipid envelopes (Table 1 and Fig. 1). Virions contain protein spikes composed of N- and C-terminal glycoprotein (G_N_, G_C_) heterodimers derived from a glycoprotein precursor (GPC). Isolated ribonucleoprotein (RNP) complexes are composed of individual segments of genomic RNA encapsidated by the nucleoprotein (N) and are associated with the large (L) protein [1].

**Table 1.** Characteristics of members of the family *Phasmaviridae*

Example	Kigluaik phantom virus (small [S] segment: KJ434184; medium [M] segment: KJ434183; large [L] segment: KJ434182), species *Orthophasmavirus kigluaikense*, genus *Orthophasmavirus*
Virion	Enveloped, spherical or pleomorphic (60–120 nm in diameter), or tubular (60×600 nm) virions with heterodimeric surface spikes
Genome	Three single-stranded RNA molecules (segments): small (S: 1.3–2.9 kb), medium (M: 2.0–6.6 kb), and large (L: 6.3–7.7 kb)
Replication	Ribonucleoprotein (RNP) complexes contain anti-genomic RNA and serve as coding templates for the synthesis of genomic RNA
Translation	Proteins are produced from capped and nonpolyadenylated mRNAs; the 5′ cap structure is obtained via cap-snatching from cellular mRNAs
Host range	Insects
Taxonomy	Realm *Riboviria,* kingdom *Orthornavirae,* phylum *Negarnaviricota*, class *Bunyaviricetes*, order *Elliovirales*: >6 genera and >28 species

## Genome

**Fig. 2.**
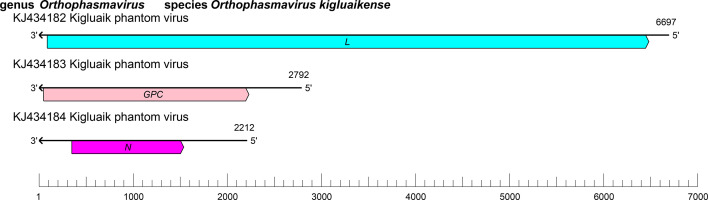
Genome organisation of Kigluaik phantom virus. *GPC*, glycoprotein precursor gene; *L*, large protein gene; *N*, nucleoprotein gene.

Phasmavirids have tri-segmented, negative-sense RNA genomes [2] (Fig. 2). These RNAs encode, in the virus-complementary sense, N (S segment), GPC (M segment), and L protein containing RdRP, helicase, and endonuclease domains (L segment). The S segment of some phasmavirids encodes a nonstructural protein (NSs) upstream of *N* [1]. Some phasmavirids also have a third ORF downstream of *N* that encodes a putative protein of unknown function [3].

## Replication

The phasmavirid lifecycle is mostly inferred from better-characterized bunyaviricetes [4] as well as jonviruses and feraviruses [1]. Infection starts with virion attachment mediated by G_N_ and G_C_. After uptake via the endosomal route, G_C_ drives membrane fusion with the host cell resulting in early or late endosomal release of the virion RNP complex into the cytoplasm in a pH-dependent manner. During primary transcription the L protein generates uncapped antigenomic RNA molecules that are then capped using host cell-derived capped primers (cap snatching). L and S segment-transcribed mRNAs are translated by free ribosomes. M segment-transcribed mRNA is translated by membrane-bound ribosomes, with the expressed GPC cleaved by cellular proteases to yield G_N_ and G_C_. Antigenomic RNA, synthesised by the L protein, serves as a template for genomic RNA replication. Secondary transcription amplifies the synthesis of mRNAs and genomic RNAs. During biogenesis, G_N_ and G_C_ traffic to the Golgi apparatus or ER-Golgi intermediate compartment and, upon interaction of their cytoplasmic tails with the viral ribonucleocapsids, new virions bud from cellular membranes for their subsequent release; specific assembly sites remain to be established.

## Taxonomy

Current taxonomy: ictv.global/taxonomy. The family *Phasmaviridae* is included in the negarnaviricot order *Elliovirales* along with crulivirids, fimovirids, hantavirids, peribunyavirids, tospovirids, and tulasvirids [5,7]. Like most other bunyaviricetes, phasmavirids (i) have multi-segmented, negative-sense RNA genomes; (ii) encode proteins with high sequence identity with those of other bunyaviricetes; (iii) have five conserved motifs (A–E) in their RdRP domain; and (iv) produce enveloped virions.

## Resources

Full ICTV Report on the family *Phasmaviridae*: www.ictv.global/report/phasmaviridae.

## Abbreviations

G_C_: C-terminal glycoprotein
G_N_: N-terminal glycoprotein
GPC: glycoprotein precursor
L: large protein
M: medium
N: nucleoprotein
NS: nonstructural protein
RdRP: RNA-directed RNA polymerase
RNP: ribonucleoprotein
S: small
